# Supplementary figures and images for: Closely related type II-C Cas9 orthologs recognize diverse PAMs
Source: eLife. 2022 Aug 12;11:e77825. doi: 10.7554/eLife.77825 (PMC9433092; doi:10.7554/eLife.77825)

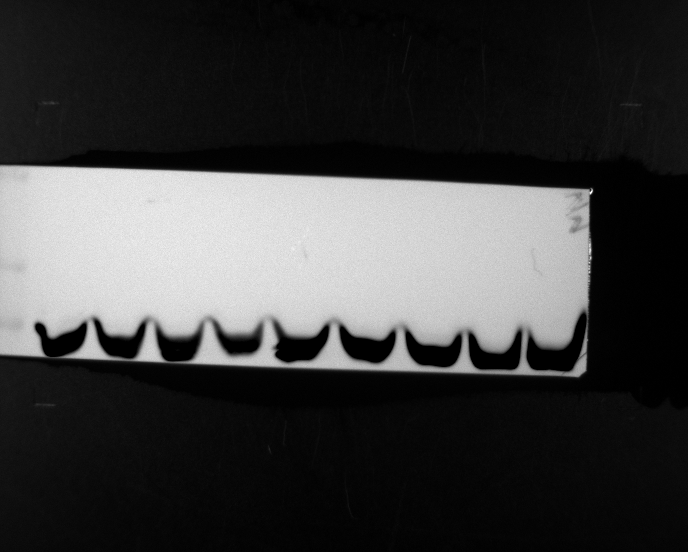

Supplement: Figure 1—figure supplement 5—source data 1. [file elife-77825-fig1-figsupp5-data1.zip › Figure 1-figure supplement 5-source data 1/full raw unedited/Nme1Cas9 orthologs-GAPDH-1.tif]

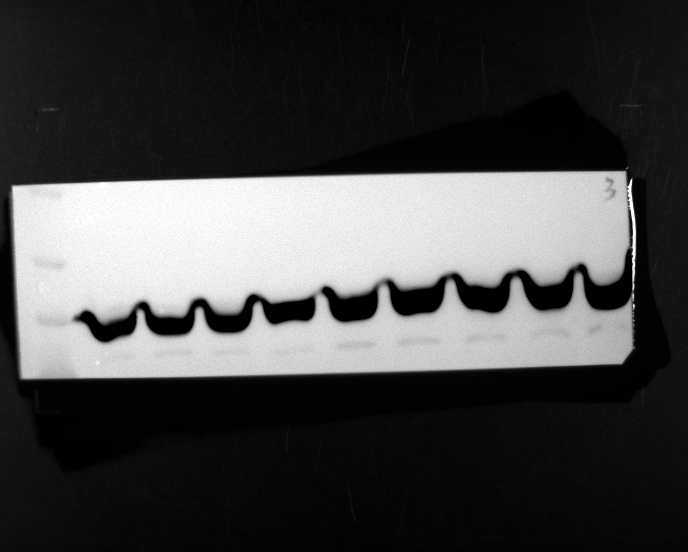

Supplement: Figure 1—figure supplement 5—source data 1. [file elife-77825-fig1-figsupp5-data1.zip › Figure 1-figure supplement 5-source data 1/full raw unedited/Nme1Cas9 orthologs-GAPDH-2.tif]

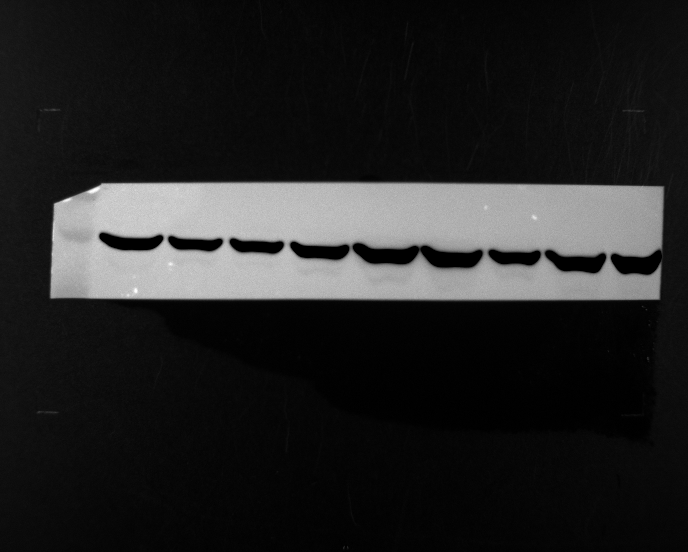

Supplement: Figure 1—figure supplement 5—source data 1. [file elife-77825-fig1-figsupp5-data1.zip › Figure 1-figure supplement 5-source data 1/full raw unedited/Nme1Cas9 orthologs-GAPDH-3.tif]

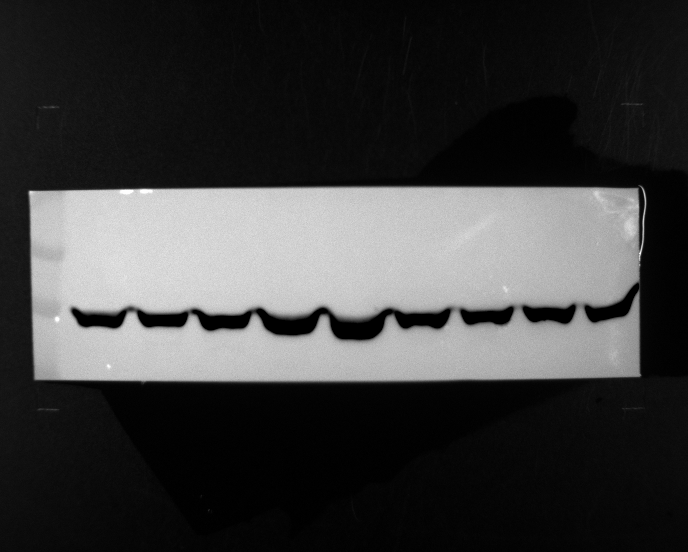

Supplement: Figure 1—figure supplement 5—source data 1. [file elife-77825-fig1-figsupp5-data1.zip › Figure 1-figure supplement 5-source data 1/full raw unedited/Nme1Cas9 orthologs-GAPDH-4.tif]

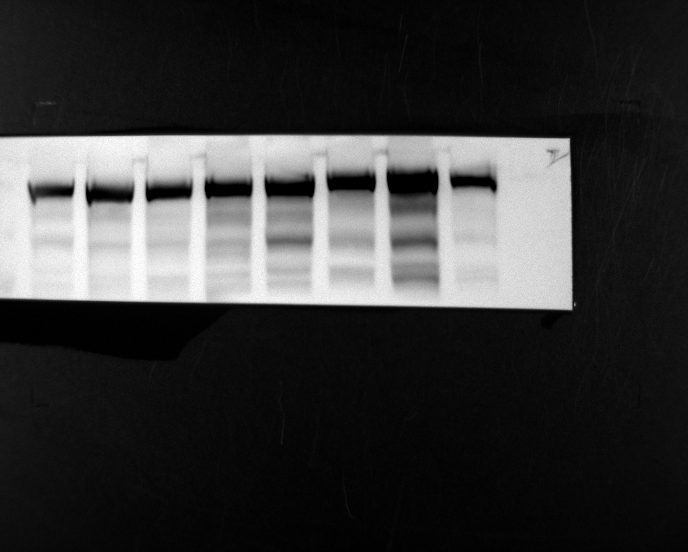

Supplement: Figure 1—figure supplement 5—source data 1. [file elife-77825-fig1-figsupp5-data1.zip › Figure 1-figure supplement 5-source data 1/full raw unedited/Nme1Cas9 orthologs-HA-1.tif]

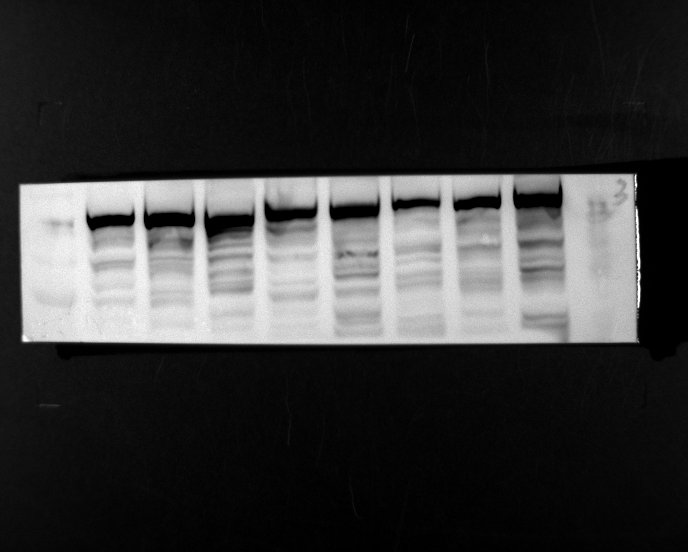

Supplement: Figure 1—figure supplement 5—source data 1. [file elife-77825-fig1-figsupp5-data1.zip › Figure 1-figure supplement 5-source data 1/full raw unedited/Nme1Cas9 orthologs-HA-2.tif]

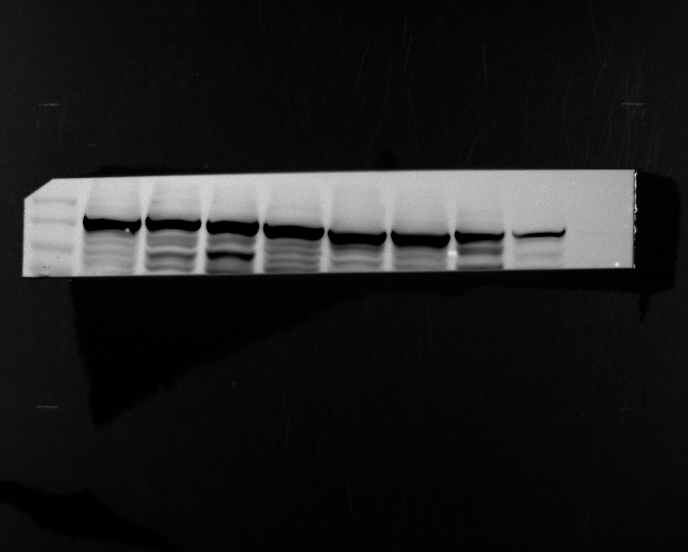

Supplement: Figure 1—figure supplement 5—source data 1. [file elife-77825-fig1-figsupp5-data1.zip › Figure 1-figure supplement 5-source data 1/full raw unedited/Nme1Cas9 orthologs-HA-3.tif]

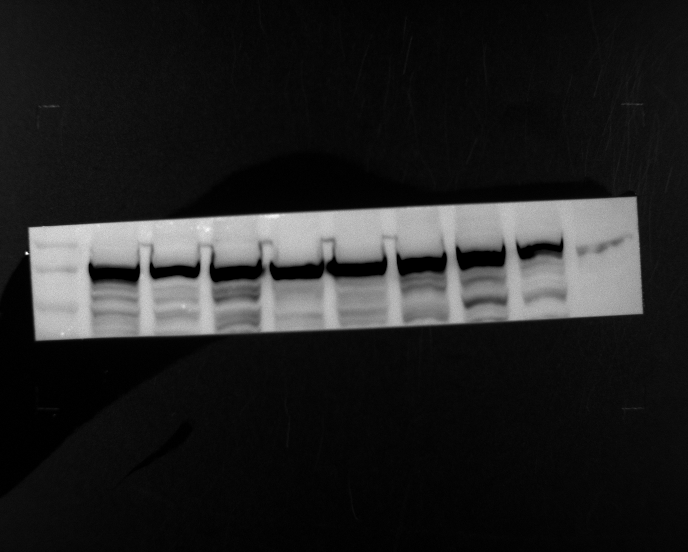

Supplement: Figure 1—figure supplement 5—source data 1. [file elife-77825-fig1-figsupp5-data1.zip › Figure 1-figure supplement 5-source data 1/full raw unedited/Nme1Cas9 orthologs-HA-4.tif]

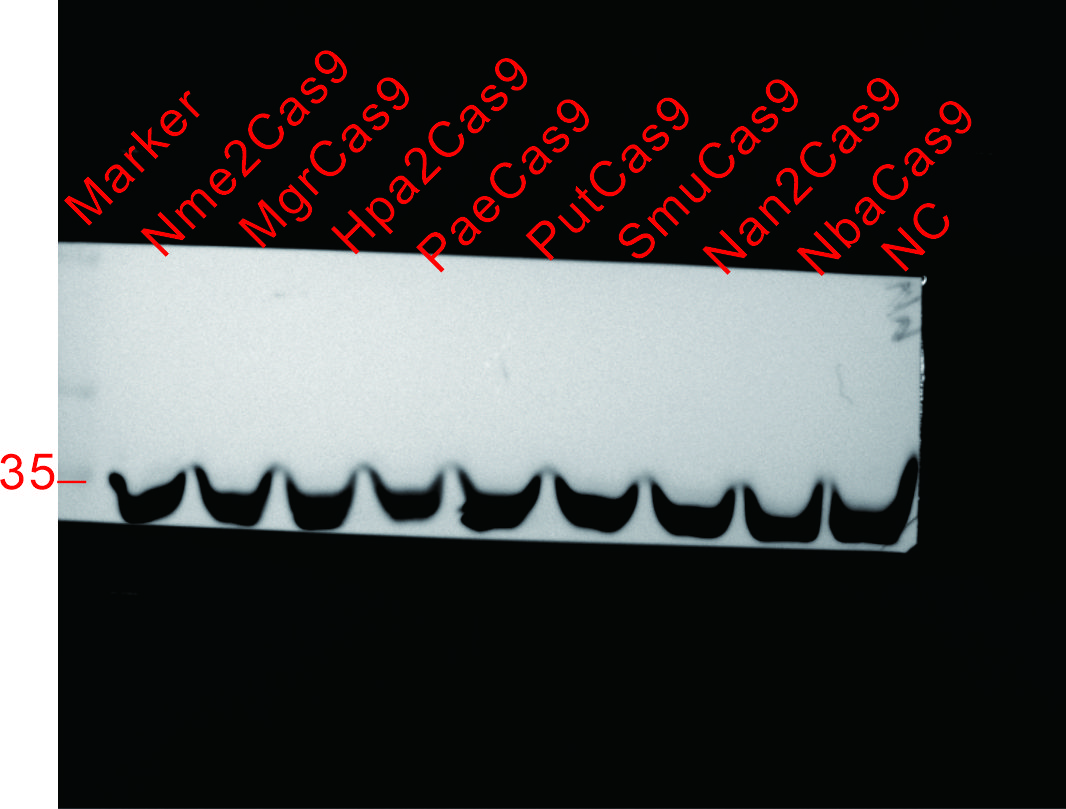

Supplement: Figure 1—figure supplement 5—source data 1. [file elife-77825-fig1-figsupp5-data1.zip › Figure 1-figure supplement 5-source data 1/Labelled/Nme1Cas9 orthologs -GAPDH-1.jpg]

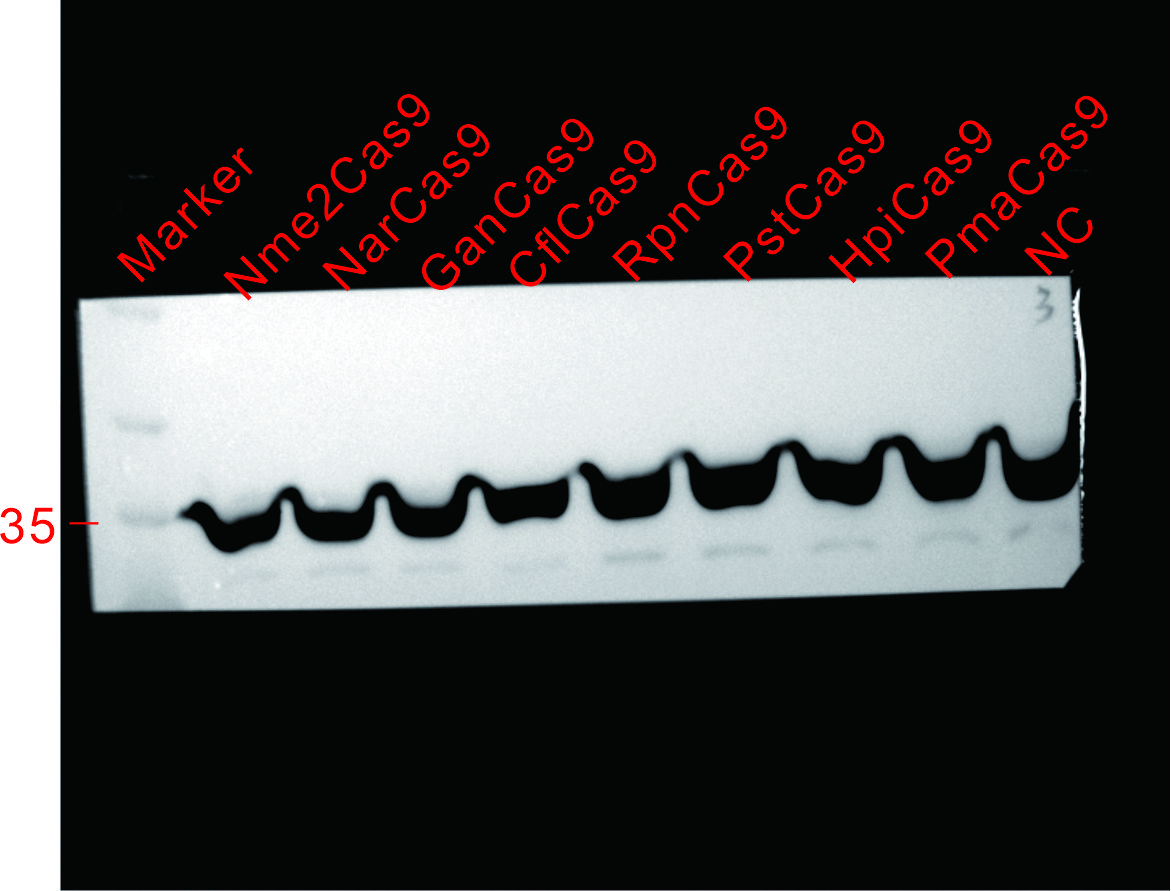

Supplement: Figure 1—figure supplement 5—source data 1. [file elife-77825-fig1-figsupp5-data1.zip › Figure 1-figure supplement 5-source data 1/Labelled/Nme1Cas9 orthologs -GAPDH-2.jpg]

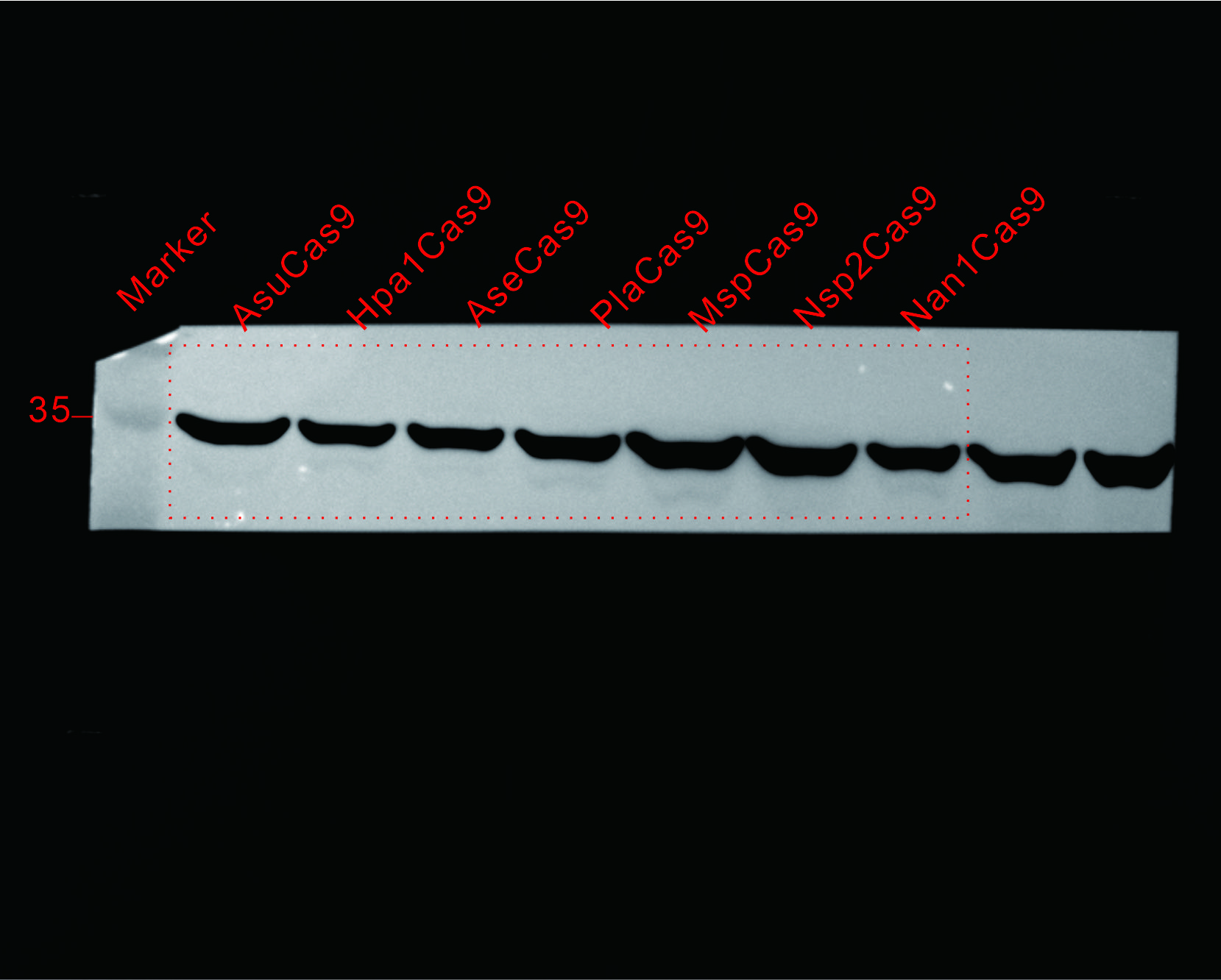

Supplement: Figure 1—figure supplement 5—source data 1. [file elife-77825-fig1-figsupp5-data1.zip › Figure 1-figure supplement 5-source data 1/Labelled/Nme1Cas9 orthologs -GAPDH-3.jpg]

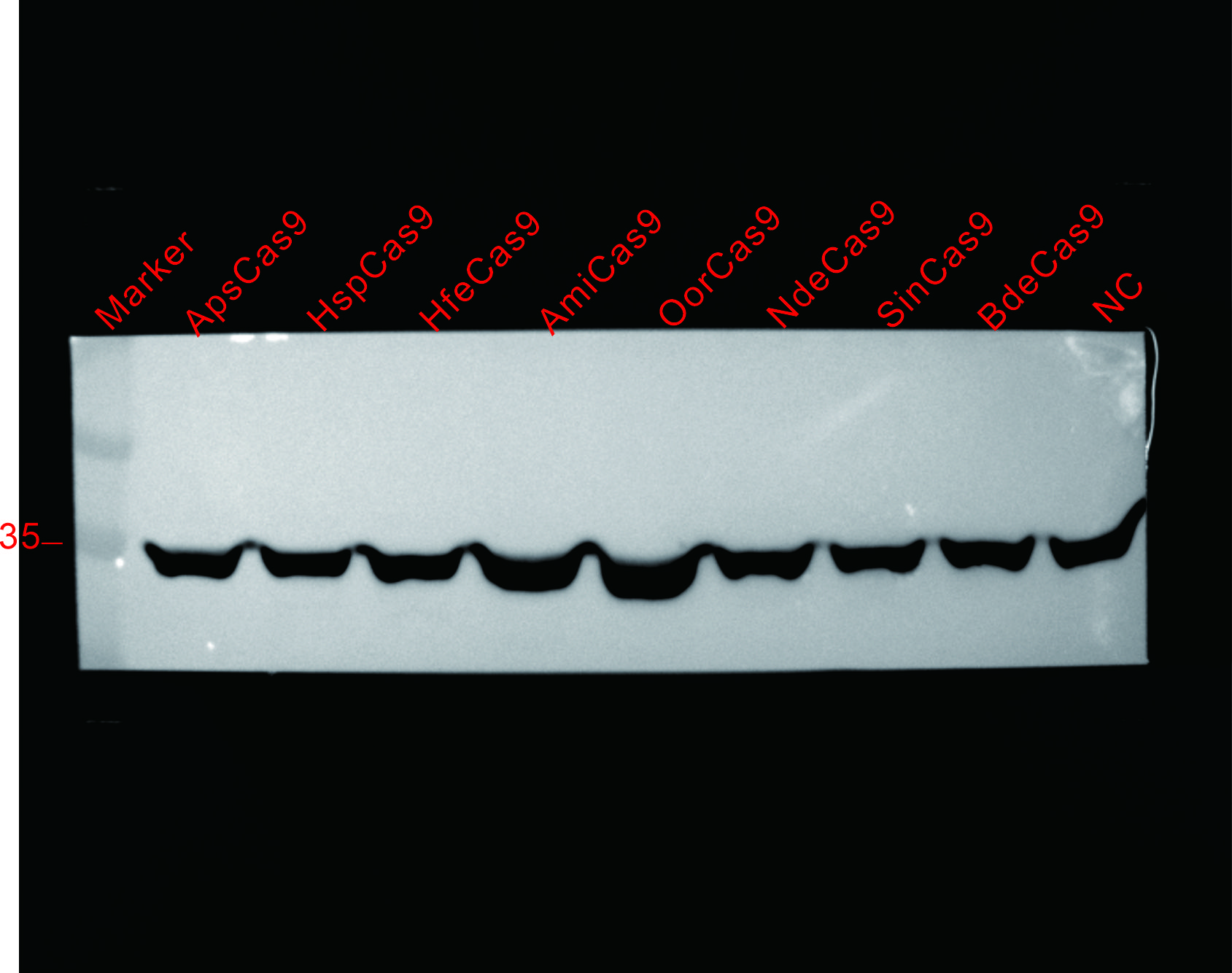

Supplement: Figure 1—figure supplement 5—source data 1. [file elife-77825-fig1-figsupp5-data1.zip › Figure 1-figure supplement 5-source data 1/Labelled/Nme1Cas9 orthologs -GAPDH-4.jpg]

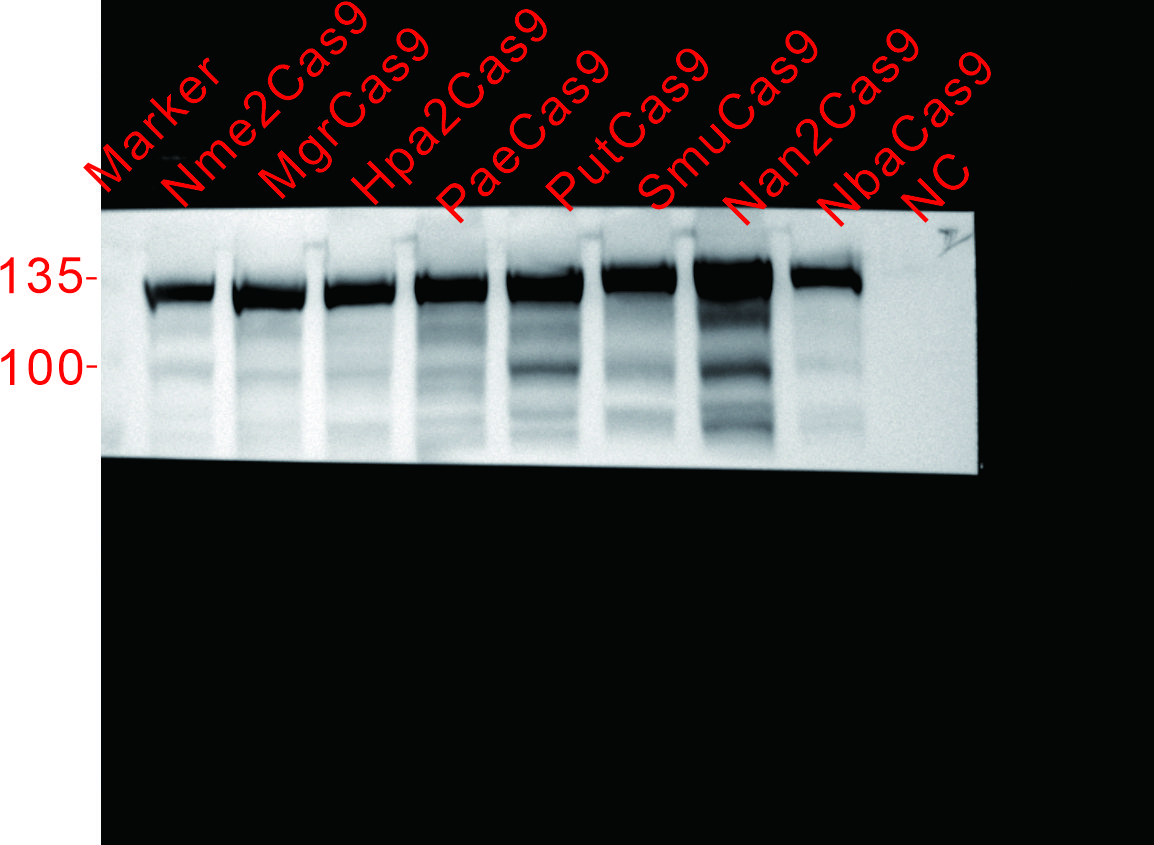

Supplement: Figure 1—figure supplement 5—source data 1. [file elife-77825-fig1-figsupp5-data1.zip › Figure 1-figure supplement 5-source data 1/Labelled/Nme1Cas9 orthologs -HA-1.jpg]

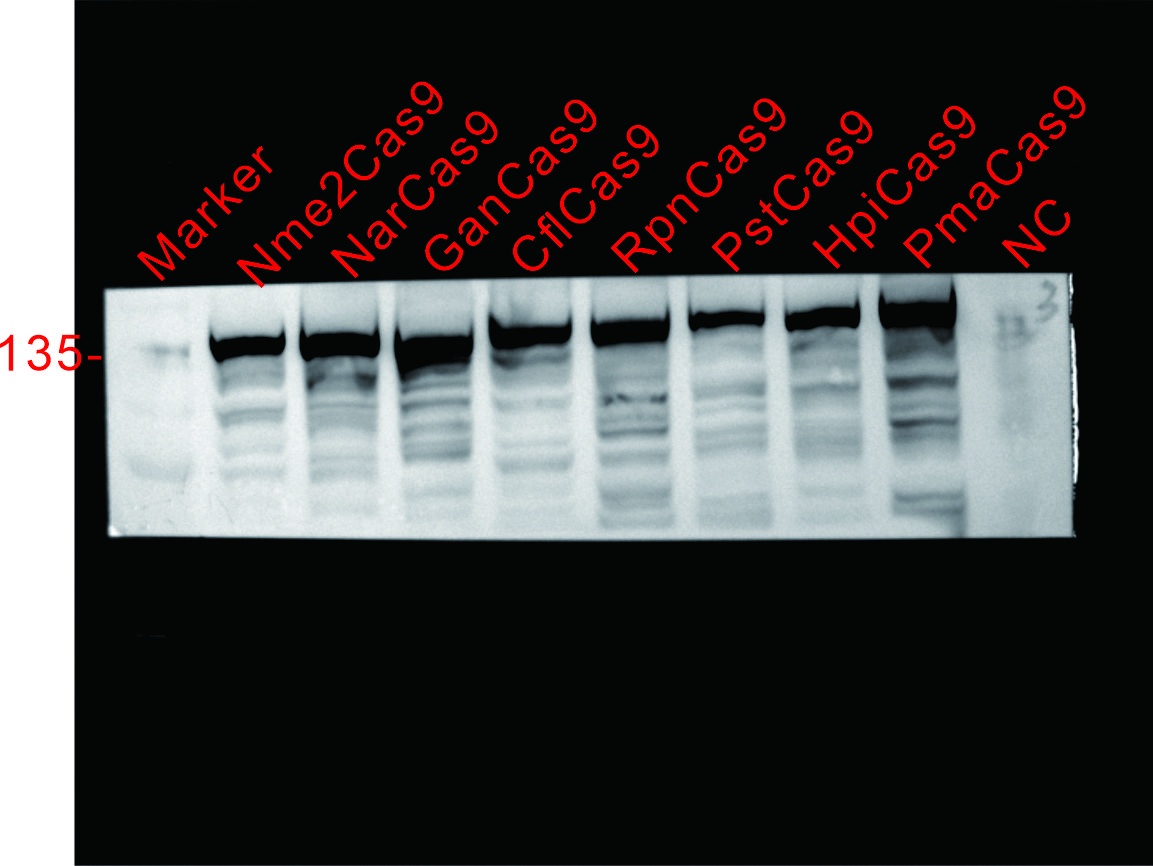

Supplement: Figure 1—figure supplement 5—source data 1. [file elife-77825-fig1-figsupp5-data1.zip › Figure 1-figure supplement 5-source data 1/Labelled/Nme1Cas9 orthologs -HA-2.jpg]

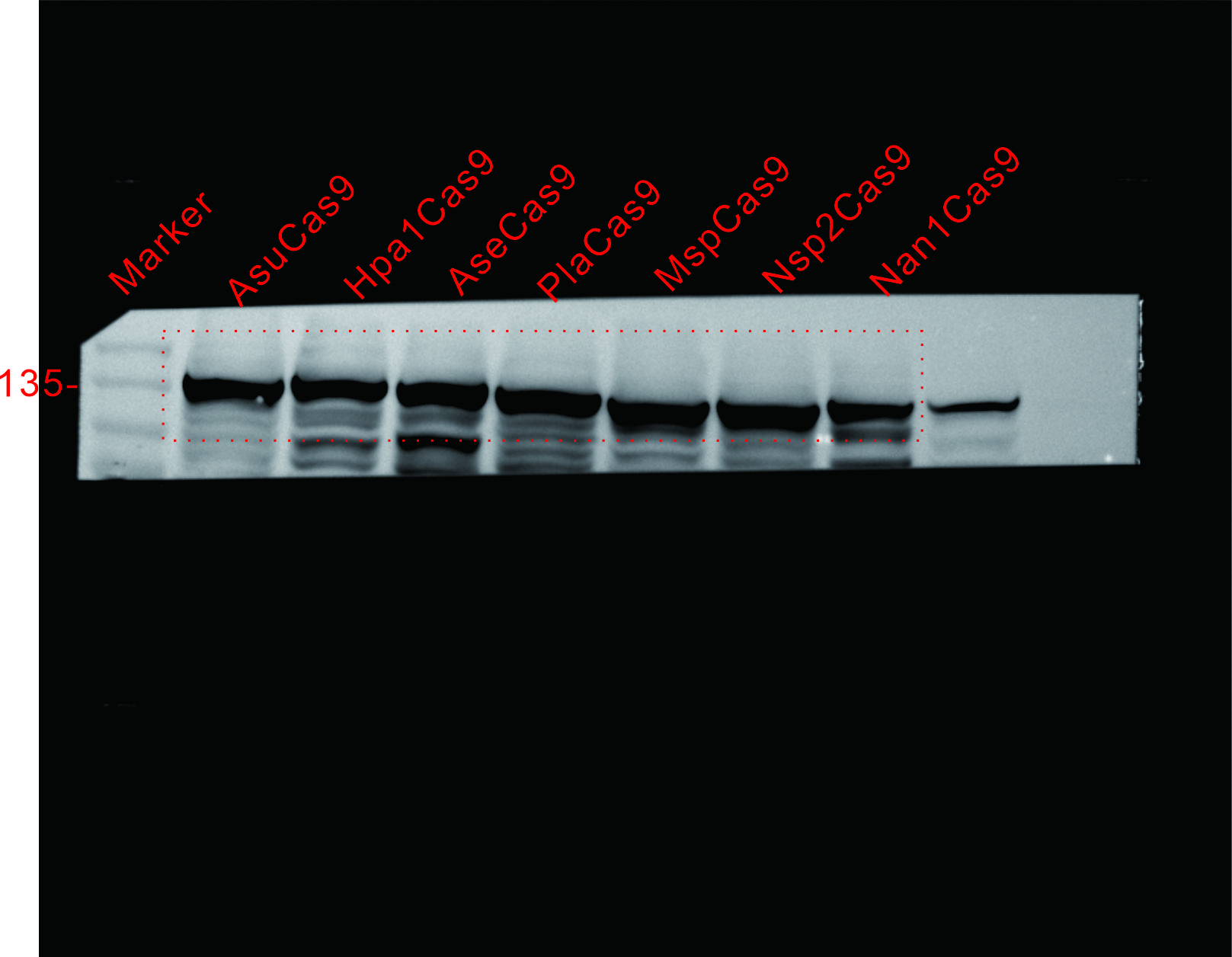

Supplement: Figure 1—figure supplement 5—source data 1. [file elife-77825-fig1-figsupp5-data1.zip › Figure 1-figure supplement 5-source data 1/Labelled/Nme1Cas9 orthologs -HA-3.jpg]

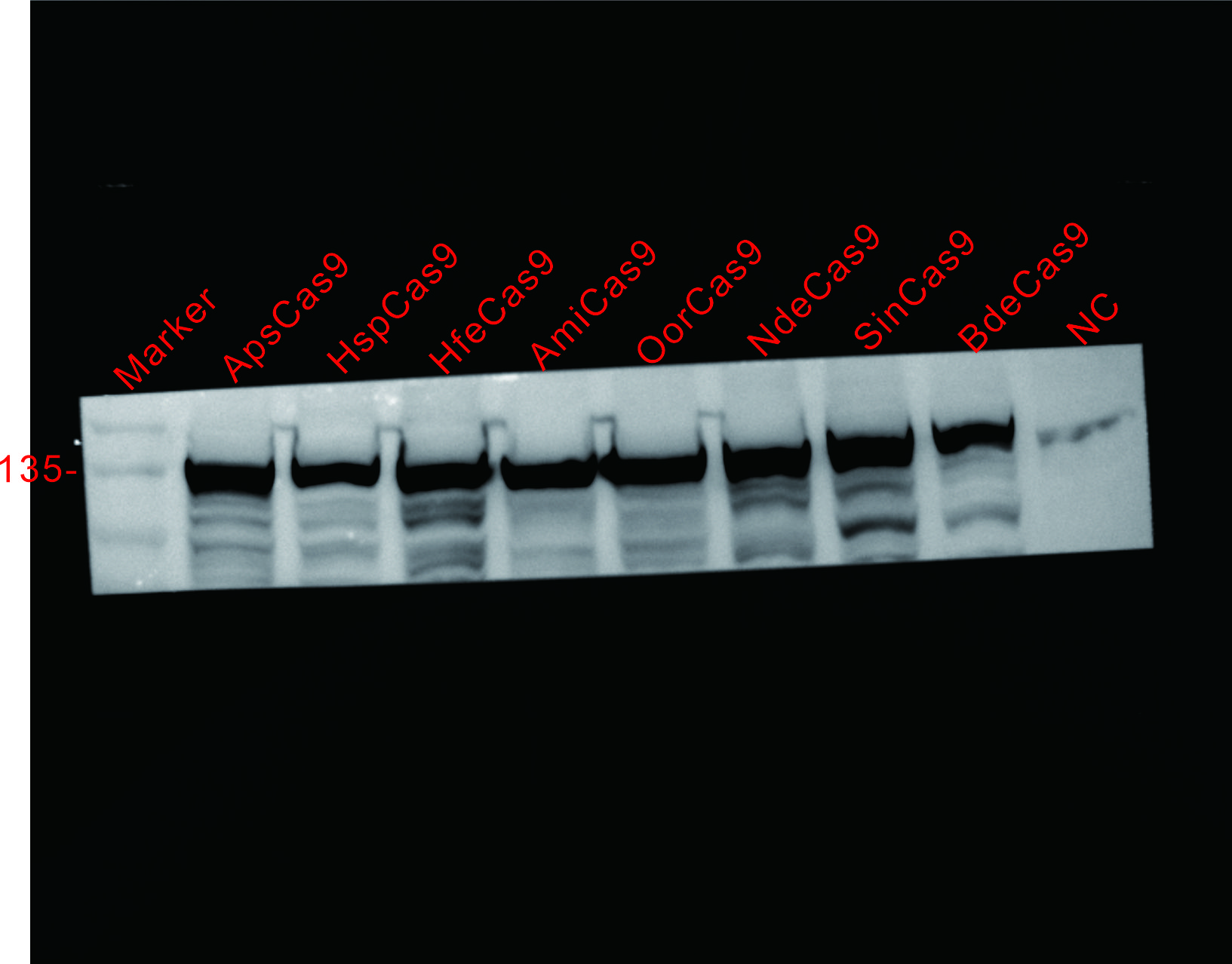

Supplement: Figure 1—figure supplement 5—source data 1. [file elife-77825-fig1-figsupp5-data1.zip › Figure 1-figure supplement 5-source data 1/Labelled/Nme1Cas9 orthologs -HA-4.jpg]

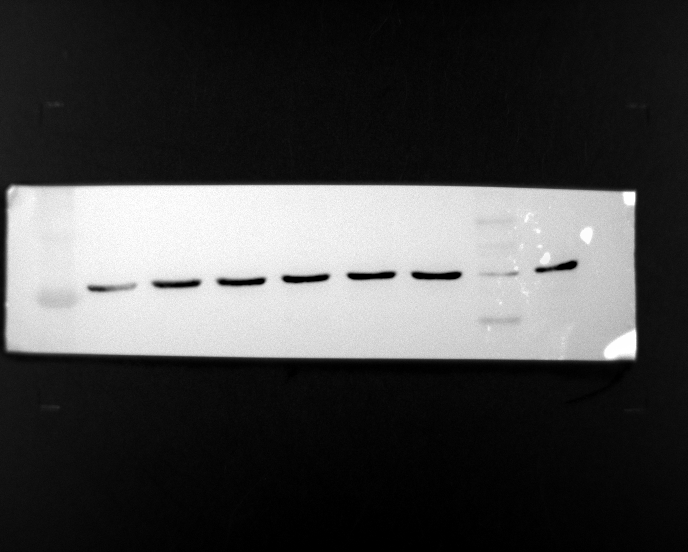

Supplement: Figure 3—source data 2. [file elife-77825-fig3-data2.zip › figure 3-source data 2/figure 3-source data 2/full raw unedited/Nsp2Cas9-gapdh-20S-M.jpg]

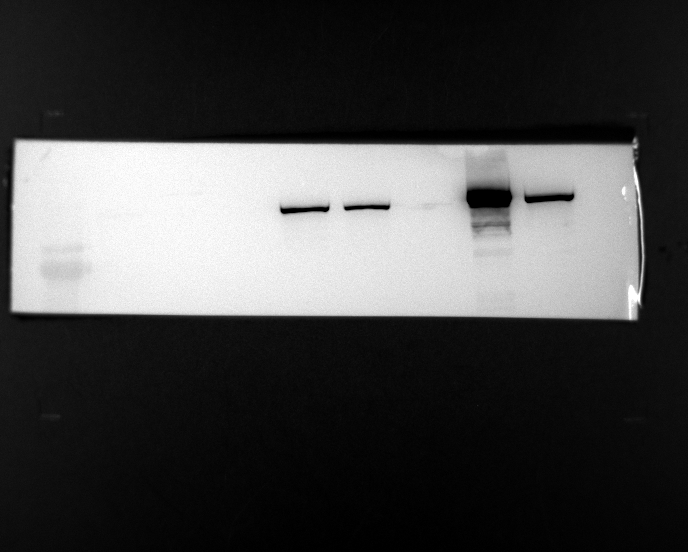

Supplement: Figure 3—source data 2. [file elife-77825-fig3-data2.zip › figure 3-source data 2/figure 3-source data 2/full raw unedited/Nsp2Cas9-HA-20S-M.jpg]

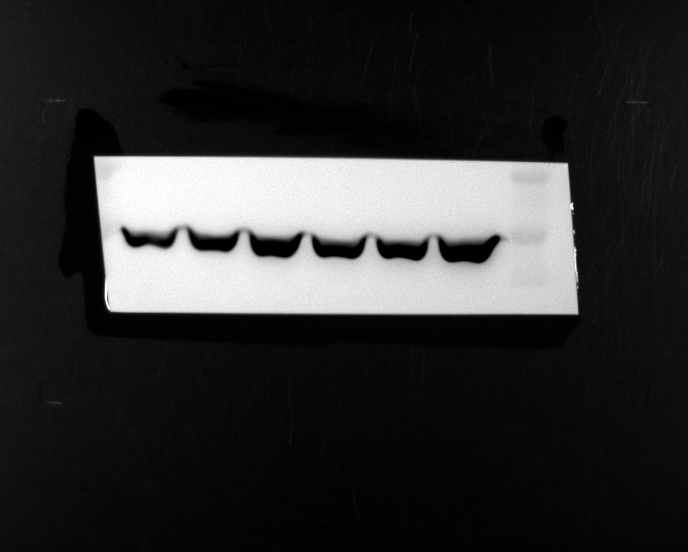

Supplement: Figure 5—source data 1. [file elife-77825-fig5-data1.zip › Figure 5-Source Data 1/full raw unedited-Nsp2-SpCas9-WB/Nsp2Cas9-SpCas9 variants-GAPDH.tif]

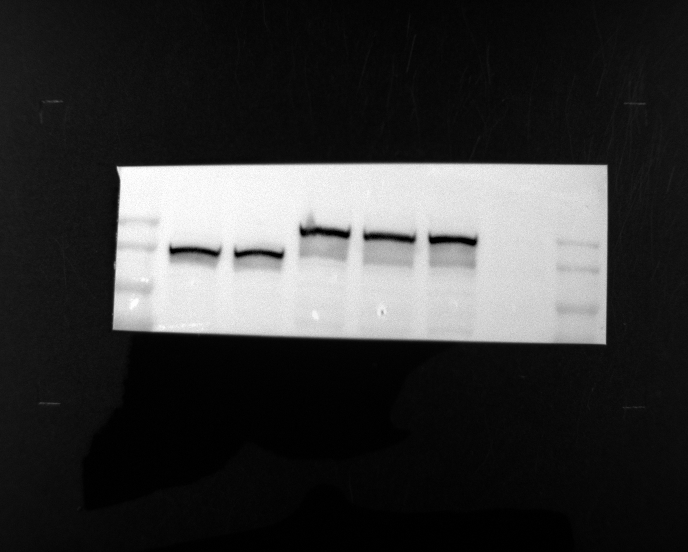

Supplement: Figure 5—source data 1. [file elife-77825-fig5-data1.zip › Figure 5-Source Data 1/full raw unedited-Nsp2-SpCas9-WB/Nsp2Cas9-SpCas9 variants-HA.tif]

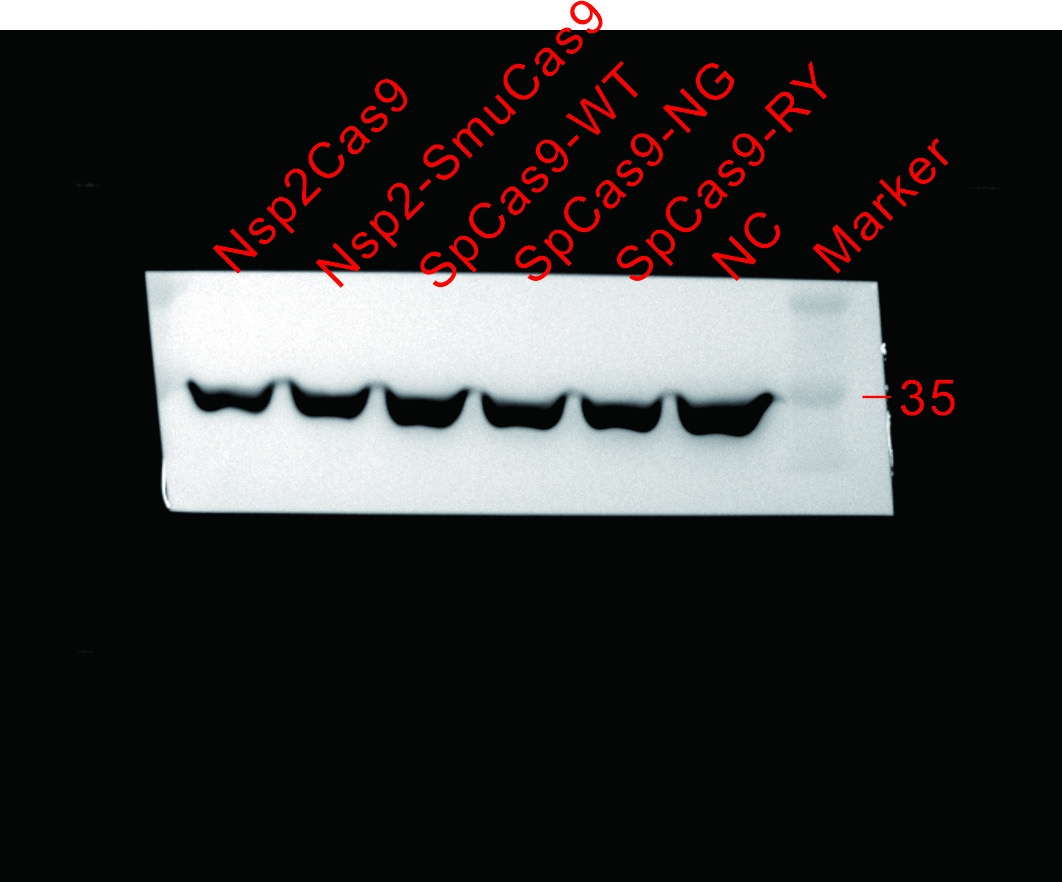

Supplement: Figure 5—source data 1. [file elife-77825-fig5-data1.zip › Figure 5-Source Data 1/labelled -Nsp2-SpCas9-WB/Nsp2-SpCas9 variants-GAPDH.jpg]

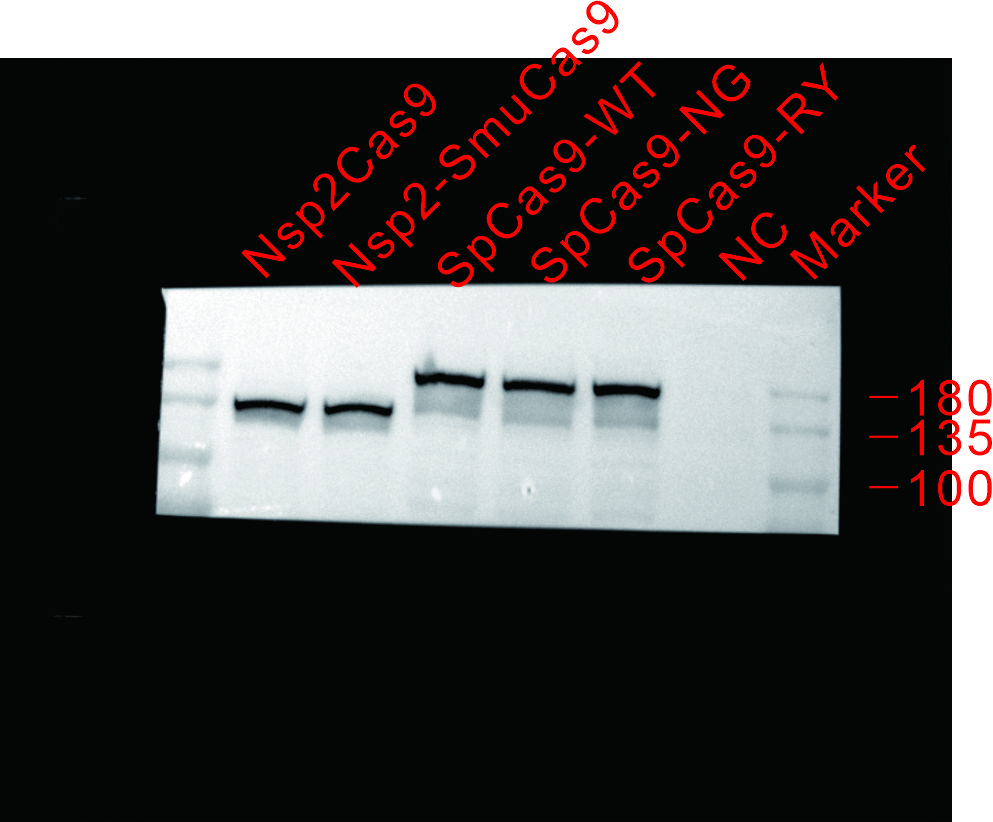

Supplement: Figure 5—source data 1. [file elife-77825-fig5-data1.zip › Figure 5-Source Data 1/labelled -Nsp2-SpCas9-WB/Nsp2-SpCas9 variants-HA.jpg]
