# Supplementary material for: Closely related type II-C Cas9 orthologs recognize diverse PAMs
Source: eLife. 2022 Aug 12;11:e77825. doi: 10.7554/eLife.77825 (PMC9433092; doi:10.7554/eLife.77825)
Supplement: Transparent reporting form [file elife-77825-transrepform1.docx]

***eLife’s* transparent reporting form**

We encourage authors to provide detailed information *within their submission* to facilitate the interpretation and replication of experiments. Authors can upload supporting documentation to indicate the use of appropriate reporting guidelines for health-related research (see [EQUATOR Network](http://www.equator-network.org/%20)), life science research (see the [BioSharing Information Resource](https://biosharing.org/)), or the [ARRIVE guidelines](http://www.plosbiology.org/article/info:doi/10.1371/journal.pbio.1000412) for reporting work involving animal research. Where applicable, authors should refer to any relevant reporting standards documents in this form.

If you have any questions, please consult our Journal Policies and/or contact us: [editorial@elifesciences.org](mailto:editorial@elifesciences.org).

**Sample-size estimation**

- You should state whether an appropriate sample size was computed when the study was being designed
- You should state the statistical method of sample size computation and any required assumptions
- If no explicit power analysis was used, you should describe how you decided what sample (replicate) size (number) to use

Please outline where this information can be found within the submission (e.g., sections or figure legends), or explain why this information doesn’t apply to your submission:

This information can be found in figure legends in our submission. Experiments with the efficiency of single gRNA were repeated at least 3 independent experiments.

**Replicates**

- You should report how often each experiment was performed
- You should include a definition of biological versus technical replication
- The data obtained should be provided and sufficient information should be provided to indicate the number of independent biological and/or technical replicates
- If you encountered any outliers, you should describe how these were handled
- Criteria for exclusion/inclusion of data should be clearly stated
- High-throughput sequence data should be uploaded before submission, with a private link for reviewers provided (these are available from both GEO and ArrayExpress)

Please outline where this information can be found within the submission (e.g., sections or figure legends), or explain why this information doesn’t apply to your submission:

All experiments of replications can be found in figure legends in our submission.

Data represent mean G SD of >3 independent experiments.

The data obtained can be found in our figure-figure supplemental-source data files.

**Statistical reporting**

- Statistical analysis methods should be described and justified
- Raw data should be presented in figures whenever informative to do so (typically when N per group is less than 10)
- For each experiment, you should identify the statistical tests used, exact values of N, definitions of center, methods of multiple test correction, and dispersion and precision measures (e.g., mean, median, SD, SEM, confidence intervals; and, for the major substantive results, a measure of effect size (e.g., Pearson's r, Cohen's d)
- Report exact p-values wherever possible alongside the summary statistics and 95% confidence intervals. These should be reported for all key questions and not only when the p-value is less than 0.05.

Please outline where this information can be found within the submission (e.g., sections or figure legends), or explain why this information doesn’t apply to your submission:

Statistical analysis methods and p-value can be found within the Material and Methods in our submission. Data represent mean G SD of 3 independent experiments. Raw data and can be found in our figure-figure supplemental-source data files.

(For large datasets, or papers with a very large number of statistical tests, you may upload a single table file with tests, Ns, etc., with reference to sections in the manuscript.)

**Group allocation**

- Indicate how samples were allocated into experimental groups (in the case of clinical studies, please specify allocation to treatment method); if randomization was used, please also state if restricted randomization was applied
- Indicate if masking was used during group allocation, data collection and/or data analysis

Please outline where this information can be found within the submission (e.g., sections or figure legends), or explain why this information doesn’t apply to your submission:

This information is not relevant to molecular/cell biology studies.

**Additional data files (“source data”)**

- We encourage you to upload relevant additional data files, such as numerical data that are represented as a graph in a figure, or as a summary table
- Where provided, these should be in the most useful format, and they can be uploaded as “Source data” files linked to a main figure or table
- Include model definition files including the full list of parameters used
- Include code used for data analysis (e.g., R, MatLab)
- Avoid stating that data files are “available upon request”

Please indicate the figures or tables for which source data files have been provided:

Figure 1-figure supplement 5-source Data 1, Figure 1-source Data 1 were submitted as source data files for the Figure 1-figure supplement 5 and Figure 1.

Figure 2-source Data 1 were submitted as source data files for the Figure 2.

Figure 3-source Data 1, Figure 3-source Data 2, Figure 3-figure supplement 1-source Data 1, Figure 3-figure supplement 2-source Data 1, Figure 3-figure supplement 3-source Data 1, Figure 3-figure supplement 4-source Data 1, and Figure 3-figure supplement 4-source Data 2 were submitted as source data files for the Figure 3, Figure 3-figure supplement 1, Figure 3-figure supplement 2, Figure 3-figure supplement 3, and Figure 3-figure supplement 4.

Figure 4-source Data 1 and Figure 4-figure supplement 1-Source Data 1 were submitted as source data files for the Figure 4 and Figure 4-figure supplement 1.

Figure 5-source Data 1 and Figure 5-source Data 2 were submitted as source data files for the Figure 5.

Figure 6-source Data 1 and Figure 7-source Data 1 were submitted as source data files for the Figure 6 and Figure 7.
